# A patient-specific timing protocol for improved CT pulmonary angiography

**DOI:** 10.1016/j.redii.2023.100036

**Published:** 2023-11-16

**Authors:** Yixiao Zhao, Logan Hubbard, Shant Malkasian, Pablo Abbona, Vijay Bosemani, Sabee Molloi

**Affiliations:** a Department of Radiological Sciences, Medical Sciences I, University of California, B-140 University of California, Irvine, CA 92697, United States

**Keywords:** Angiography, Computed tomography, Contrast media, Pulmonary embolism

## Abstract

**Rationale and objectives:**

To improve the image quality of CT pulmonary angiography (CTPA) using a patient-specific timing protocol.

**Material and methods:**

A total of 24 swine (48.5 ± 14.3 kg) underwent continuous contrast-enhanced dynamic CT acquisition over 30 s to capture the pulmonary arterial input function (AIF). Multiple contrast injections were made under different cardiac outputs (1.4–5.1 L/min), resulting in a total of 154 AIF curves. The volume scans with maximal enhancement in these AIF curves were retrospectively selected as the reference standard (group A). Two prospective CTPA protocols with bolus-tracking were then simulated using these AIF curves: one used a fixed delay of 5 s between triggering and CTPA acquisition (group B), while the other used a specific delay based on one-half of the contrast injection duration (group C). The mean attenuation, signal-to-noise (SNR) and contrast-to-noise ratios (CNR) between the three groups were then compared using independent sample *t*-test. Subjective image quality scores were also compared using Wilcoxon-Mann-Whitney test.

**Results:**

The mean attenuation of pulmonary arteries for group A, B and C (expressed in [HU]) were 870.1 ± 242.5 HU, 761.1 ± 246.7 HU and 825.2 ± 236.8 HU, respectively. The differences in the mean SNR and CNR between Group A and Group C were not significant (SNR: 65.2 vs. 62.4, CNR: 59.6 vs. 56.4, both *p* > 0.05), while Group B was significantly lower than Group A (*p* < 0.05).

**Conclusion:**

The image quality of CT pulmonary angiography is significantly improved with a timing protocol determined using contrast injection delivery time, as compared with a standard timing protocol with a fixed delay between bolus triggering and image acquisition.

## 1 Introduction

Computed tomography pulmonary angiography (CTPA) is the current standard imaging modality in the diagnosis and assessment of suspected pulmonary embolism (PE) [1], [2], [3], [4], [5], [6]. While newer generation CT scanners have improved both temporal and spatial resolutions for CTPA and CT perfusion, existing standard acquisition protocols do not consistently acquire images at the peak of arterial contrast enhancement, leading to suboptimal image quality and reduced diagnostic accuracy [7], [8], [9], [10], [11]. Hence, the optimization of CTPA contrast timing to provide adequate opacification of the pulmonary artery and its branches remains highly relevant [2,12,13]. Consequently, an accurate, robust timing protocol for patient-specific CTPA acquisition protocol that could be used for patients with varying hemodynamic states is essential [8,14,15].

Test bolus technique has been used to predict the proper scan timing of CTPA [16,17]. While the test bolus technique was shown to improve the prediction of contrast arrival time to the pulmonary circulation, it is difficult to accurately predict the CTPA acquisition time at the peak of the pulmonary contrast enhancement because the amount of contrast material used for a test bolus is usually much smaller than used for the diagnostic CTPA [18]. Moreover, the need for additional contrast material, radiation dose, and the necessary manual steps of operator interactions makes the test bolus less optimal. Alternatively, the bolus-tracking technique provides a better solution and has become the clinical standard for CTPA [8,[17], [18], [19], [20], [21], [22], [23], [24]]. Specifically, bolus tracking enables automated real-time monitoring of the contrast attenuation inside the sampling arterial vessel of interest for CTPA triggering. However, the optimal time delay between bolus tracking trigger and acquisition of CTPA at peak enhancement remains unknown. Existing standard CTPA protocols employ a fixed delay of 4–7 s without considering patient-specific parameters such as contrast injection protocol and hemodynamic conditions. A patient-specific delay time between bolus tracking trigger and image acquisition could potentially improve image quality and reduce the necessary contrast volume.

Hence, the purpose of this preclinical swine study was to assess the objective and subjective image quality of CTPA with a patient-specific delay time between bolus tracking trigger and image acquisition as compared to a standard fixed delay time, using 20 volume scans acquired for CTPA as the reference standard. The patient-specific delay time was determined by one-half of contrast injection duration plus a constant dispersion delay [25]. The central hypothesis was that the patient-specific delay time can yield images with higher contrast-to-noise ratio and better diagnostic image quality as compared to the standard protocol with a fixed delay time.

## 2 Material and methods

### 2.1 General methods

The study was approved by the Institutional Animal Care and Use Committee (IACUC, Protocol Number: AUP-18–191) at University of California. Twenty-four male Yorkshire swine (48.5±14.3kg) were used for the study. Following contrast injection, continuous CT data were acquired in all animals. Using the continuous datasets, bolus-tracking with two different acquisition protocols was then simulated: a standard protocol with a fixed-delay between triggering and CTPA acquisition (group B), and a patient-specific protocol with a variable delay (group C) determined using one-half of the contrast injection duration plus a predefined constant dispersion factor. The objective and subjective image qualities of the two prospective CTPA protocols were then compared to the maximal contrast attenuation of the continuous datasets (group A) as the reference standard. All experimental data were acquired between March 2016 and December 2017 and were analyzed between June 2018 and June 2020. All authors participated in the experimental design and data acquisition. YZ, LH and ShM had more than three years of medical imaging research experience and conducted the data analysis. PA and VB were two radiologists with more than 15 years of clinical experience, helped with clinical evaluation of CTPA image quality.

### 2.2 Animal preparation

Anesthesia was induced with telazol (4.4 mg/kg), ketamine (2.2 mg/kg) and xylazine (2.2 mg/kg), intubated (Mallinckrodt, tube 6.0–8.0 mm, Covidien, Mansfield, MA) and mechanically ventilated (Surgivet, Norwell, MA, and Highland Medical Equipment, Temecula, CA). Anesthesia was maintained with 1.5%−2.5% isoflurane (Baxter, Deerfield, IL) and oxygen throughout the entire experiment. Catheters (5-Fr AVANTIR, Cordis Corporation, Miami Lakes, FL) were placed in the right femoral vein, left femoral vein and right femoral artery for intravenous contrast injection, fluid administration and pressure monitoring, respectively. Vital signs such as electrocardiogram (ECG), mean arterial pressure (mmHg), end-tidal CO_2_ (mmHg), and O_2_ saturation (%) were monitored. At the end of each experiment, each animal was euthanized with saturated KCI under deep anesthesia.

### 2.3 CT imaging protocol

Each swine was imaged with a 320-slice CT scanner (Aquilion One, Canon America Medical Systems, Tustin, CA). First, a scout scan was obtained to correctly position the 16-cm craniocaudal field-of-view for pulmonary arterial imaging. Iodinated contrast agent (Isovue 370, Bracco Diagnostics, Princeton, NJ) was then injected (0.5–1 ml/kg) at 5 ml/s followed by a saline chaser (0.25–0.5 ml/kg) at the same rate (Empower CTA, Acist Medical Systems, Eden Prairie, MN). Twenty ECG-gated volume scans were then acquired continuously during an inspiratory breath-hold of 30 s. ECG gating was used to minimize blurring artifacts close to the heart region. Five to eight contrast injections were made under various cardiac outputs due to variations over time following relatively high volume of contrast material and anesthesia with at least 15 min between injections, resulting in a total of 154 acquisitions for all the animals. Images were acquired at 100 kV and 200 mA, with a detector collimation of 320 × 0.5 mm and a gantry rotation time of 0.35 s. The scan and reconstructed field-of-views were in the range of 240 to 400 mm. All images were reconstructed using a soft tissue kernel (FC07) with a 0.5-mm slice thickness and an adaptive iterative dose reduction 3D reconstruction algorithm.

### 2.4 Image analysis and reference standard CTPA

The dynamic volumes were registered to a single coordinate system by applying a GPU-based affine and deformable registration algorithm (22). A region-of-interest (ROI) was then placed in the main pulmonary artery of each acquisition to generate the pulmonary arterial input function (AIF). In each case, the image volume at the peak attenuation of the AIF was retrospectively selected and defined as the reference standard CTPA (group A).

### 2.5 Standard and patient-specific timing protocols

**Fig. 1:**
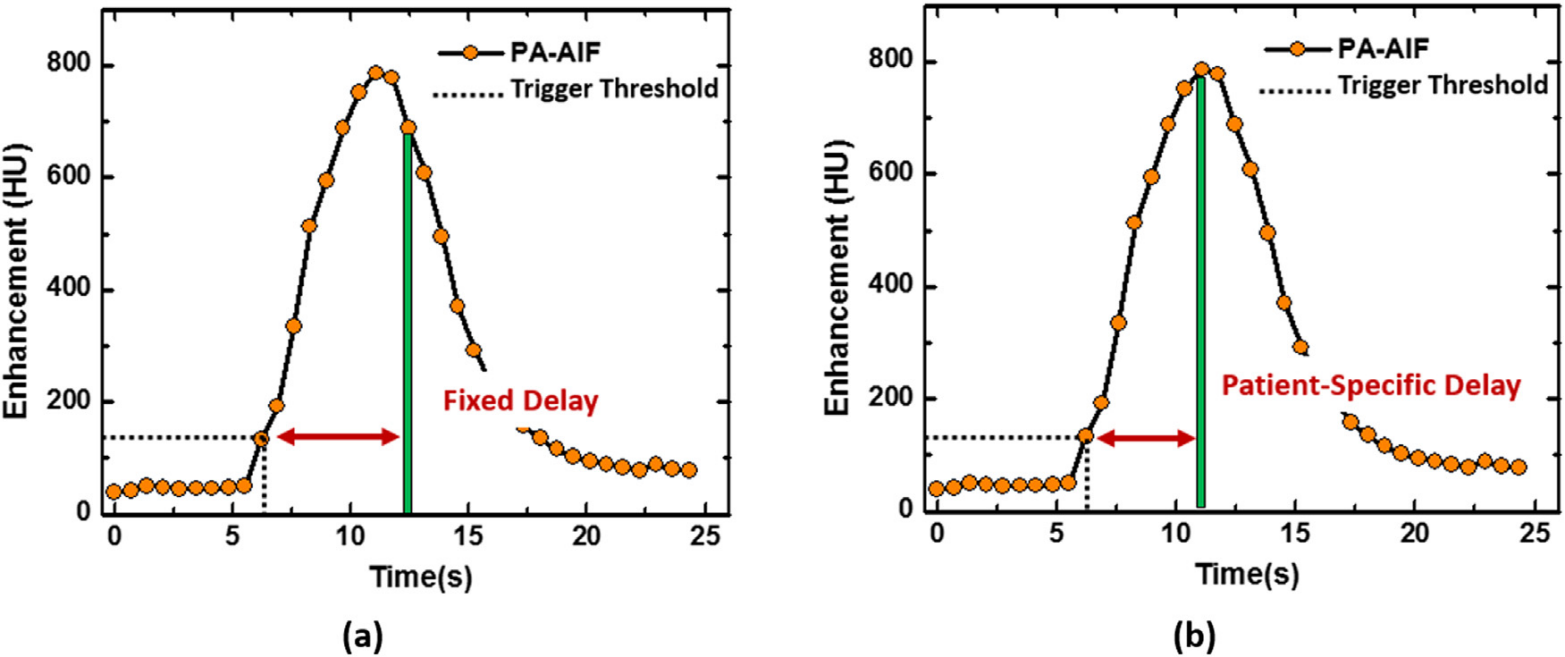
Prospective protocols for (a) standard fixed-delay timing protocol and (b) patient-specific timing protocol. The arterial input function in the pulmonary artery trunk (PA-AIF) is shown and used to simulate bolus-tracking. For (a), the CTPA volume scan is acquired 5 s after the triggering. For (b), the CTPA volume scan is acquired after a patient-specific time delay. The patient-specific delay is derived from the one-half of injection duration plus a dispersion delay.

For each acquisition dataset, two different CTPA protocols were simulated. In each case, the AIF was used for the simulation of bolus-tracking triggering. Multiple contrast injections were made in each animal that gradually increases the intensity of the blood pool so a single bolus tracking trigger threshold could not be used for all the studies. Therefore, the bolus tracking ‘trigger’ was calculated for each study by adding 60 HU to the baseline blood pool HU. The baseline blood pool HU was calculated by averaging the ROIs from the first three non-enhanced images of the AIF curve. For standard protocol (group B), the CTPA ‘acquisition’ occurred 5 s after the triggering time point (Fig. 1a). For patient-specific protocol (group C), the CTPA ‘acquisition’ occurred after a patient-specific delay time that was estimated using one-half of contrast injection duration and a constant dispersion delay (Fig. 1b) [18,25]. Specifically, given a fast injection rate and short injection duration, the contrast bolus would be diluted into a gaussian distribution, where the maximal enhancement located at the center of the contrast pass curve and such time-to-peak delay can be related to one-half of contrast injection duration. The dispersion delay is a constant describing the dispersion of the contrast bolus, which is determined by the distance between the bolus tracking ROI and contrast injection site. For the ROI within the pulmonary artery, the dispersion delay is approximately one second from a previous study.

### 2.6 Objective image quality assessment

To assess the level of arterial contrast enhancement achieved by each protocol, ROIs were placed in the main pulmonary artery (MPA), right pulmonary artery (RPA), left pulmonary artery (LPA), segmental arteries of the right upper lobe (RUL), right middle lobe (RML), right caudal lobe (RCL), left upper lobe (LUL), left lingula lobe (LLL), left caudal lobe (LCL), accessory lobe (AL), and sub-segmental arteries of the right caudal lobe (RCL_S) and left caudal lobe (LCL_S). In each case, the ROI was manually defined as a circular region ranging in size from 9 cm^2^ for the MPA to 0.3 cm^2^ for the sub-segmental arteries. The mean contrast enhancement within each vessel ROI was then calculated for group A, B and C. The signal-to-noise (SNR) and the contrast-to-noise (CNR) were also computed in each case. Specifically, three additional ROIs (∼3 cm^2^) were drawn in artifact-free air region (anterior, left, and right, outside of the swine) and the average standard deviation in HU was defined as the background noise. The SNR of each vascular ROI was then determined as the ratio of the ROI contrast enhancement to the background noise. Two final ROIs (∼2 cm^2^) were then placed in the paraspinal and subscapular muscles, and the average muscular contrast enhancement was determined. The CNR of each vascular ROI was then determined as the difference between the vascular and muscular contrast enhancement divided by the image noise.

### 2.7 Subjective image quality assessment

Two independent blinded readers (P.A and V.B) assessed the overall subjective image quality of the pulmonary arteries for group A, B, and C, with a five-point Likert scale (10) (1 = excellent, 2 = good, 3 = adequate, 4 = poor, 5 = non-diagnostic quality). Prior to reading the images from this study, 15 external CTPA images were first rated with the Likert score and discussed between the two readers. In the case of large disagreements between readers (score difference ≥ 3), the quality score was reevaluated and with the final score determined by a second joint-reading round.

### 2.8 Radiation dose

For each acquisition, the CT dose index (${\text{CTDI}}_{\text{vol}}^{32}$, mGy) and the dose-length product (DLP, mGy·cm) were recorded from the scanner dose summary log. Additionally, the effective diameter (ED) of each swine was calculated using the anteroposterior diameter (AD) and lateral diameter (LD) of the chest window ($\text{ED}=\sqrt{\text{AD}\times \text{LD}}$). Size-specific dose estimates (SSDE, mGy) were then calculated using a size-specific conversion factor [26].

### 2.9 Statistical approach

All analyses were performed using IBM SPSS Statistics software (Version 22.0; Armonk NY). Quantitative variables were compared with mean ± standard deviation (SD) and 95% confidence intervals (CI). All objective and subjective image quality parameters were first verified to be normally distributed using skewness and kurtosis values and the Shapiro-Wilk test. The CT attenuations, SNR and CNR of all measurement vessels were then compared by independent-samples t tests. Additionally, the mean attenuations of different vessel generations were compared with Box-and -Whisker plot. In each case, a two-tailed p-value less than 0.05 was considered to be statistically significant. Subjective image quality was assessed between the two readers by rating the 12 pulmonary artery locations where the average score was calculated for each observer. The differences in rating scores across the study groups were then tested by the Wilcoxon-Mann-Whitney test. The interobserver agreement of subjective image quality was evaluated with Cohen kappa value (*k*) with quadratic weighting [27]. The kappa values were interpreted as: < 0.20, poor agreement; 0.21–0.40, fair agreement; 0.41– 0.60, moderate agreement, 0.61–0.80, good agreement, and 0.81–1.00, excellent agreement [27].

## 3 Results

### 3.1 Vitals and CT radiation dose

Twenty-four swine were used in the study (body weight: 48.48 ± 14.33 kg, heart rate 89.50 ± 14.96 bpm) with 5–8 contrast injections for each animal, resulting in a total of 154 independent contrast enhanced CT acquisitions. The average dose for the continuous scan was 258.2mGy. For the simulated prospective CTPA acquisitions, the ${\text{CTDI}}_{\text{vol}}^{32}$ was calculated to be 9.4 mGy, and the SSDE was estimated to be 17.8 mGy. The cardiac output for each acquisition was estimated by the whole-lung blood flow using a well-established CT pulmonary perfusion measurement [25,28]. The overall cardiac output was in a range of 1.4–5.1 L/min.

### 3.2 Subjective assessment

Overall subjective image quality between the two readers was in good agreement (Cohen quadratic weight *k* = 0.64, 95% CI: [0.47, 0.81]), as displayed in Table 1. There were no significant differences in image quality scores overall between the two readers (*p* = 0.89). The largest disagreements were found between excellent (84.0% vs. 65.1%) and good (10.8% vs. 26.0%) image quality scores, while the maximum difference in image quality scores between readers was two points. The Likert scores from both readers were averaged and the group A, B, and C were compared. Significant differences were found between all three groups (*p* < 0.001), where the average subjective image quality score for each group was 1.24 ± 0.42, 1.46 ± 0.78 and 1.35 ± 0.59, respectively.

**Table 1:** Subjective image quality assessment.

	GROUP A (*N*=154)		GROUP B (*N*=154)		GROUP C (*N*=154)		OVERALL (*N*=452)		
	Observer 1	Observer 2	Observer 1	Observer 2	Observer 1	Observer 2	Observer 1	Observer 2	Both Observers
1	141 (91.6%)	106 (68.9%)	119 (77.2%)	96 (62.3%)	128 (83.1%)	99 (64.3%)	388 (84.0%)	301 (65.1%)	290 (62.8%)
2	9 (5.8%)	40 (26.0%)	23 (14.9%)	39 (25.3%)	18 (11.7%)	40 (26.0%)	50 (10.8%)	119 (26.0%)	136 (29.4%)
3	4 (2.6%)	8 (7.8%)	5 (3.2%)	13 (8.4%)	7 (4.5%)	14 (9.1%)	16 (3.5%)	35 (7.6%)	28 (6.06%)
4	0 (0.0%)	0 (0.0%)	4 (2.6%)	3 (1.3%)	0 (0.0%)	1 (0.6%)	4 (0.9%)	4 (0.9%)	4 (0.9%)
5	0 (0.0%)	0 (0.0%)	3 (1.9%)	3 (1.9%)	1 (0.6%)	0 (0.0%)	4 (0.9%)	3 (0.7%)	4 (0.9%)
Kappa value	0.37		0.74		0.62		0.64		
95% CI	[nc, nc]		[0.62, 0.87]		[0.28, 0.96]		[0.47, 0.81]		

Note.- Group A: actual peak image from the retrospective arterial input function. Group B: fixed-delay CTPA image. Group C: patient-specific-delay CTPA image. The kappa values showed in the table are quadratic weighted values. CI: confidence interval. NC: The quantity cannot be calculated. This occurred when the data entries include a substantial proportion of zeros. 1 = excellent, 2 = good, 3 = adequate, 4 = poor, 5 = non-diagnostic quality.

**Fig. 2:**
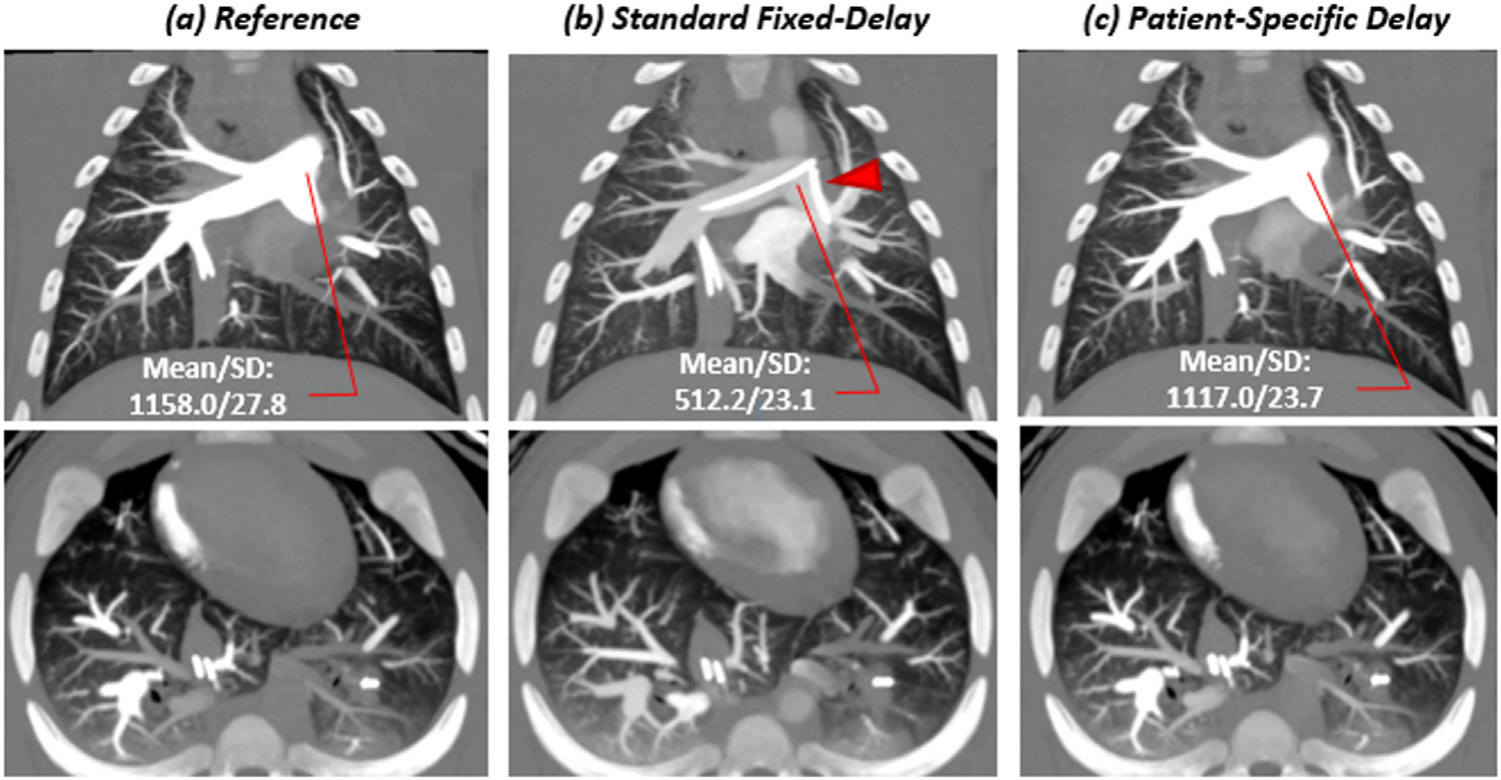
Images for a 27kg swine with an injection of 0.5 ml/kg contrast at a rate of 5 ml/s under balloon occlusion. Contrast material–enhanced CT angiography (a) using retrospective peak from the pulmonary arterial input function (AIF); (b) with standard fixed-delay protocol; (c) with patient-specific timing protocol in a 10mm maximum-intensity projection (MIP) image from both coronal and axial rendering ([*window*/*level*: 1600/500]. In the main pulmonary artery, The CT numbers for (a), (b) and (c) were 1158 HU, 512.2 HU and 1170 HU; the SNR were 134.1, 60.1 and 131.0; the CNR were 126.5, 50.9 and 121.9, respectively. For subjective image quality the scores were 1, 1, 1 respectively for reader 1, and were 1,2, 1 respectively for reader 2. Red arrows indicate the balloon catheter.

**Fig. 3:**
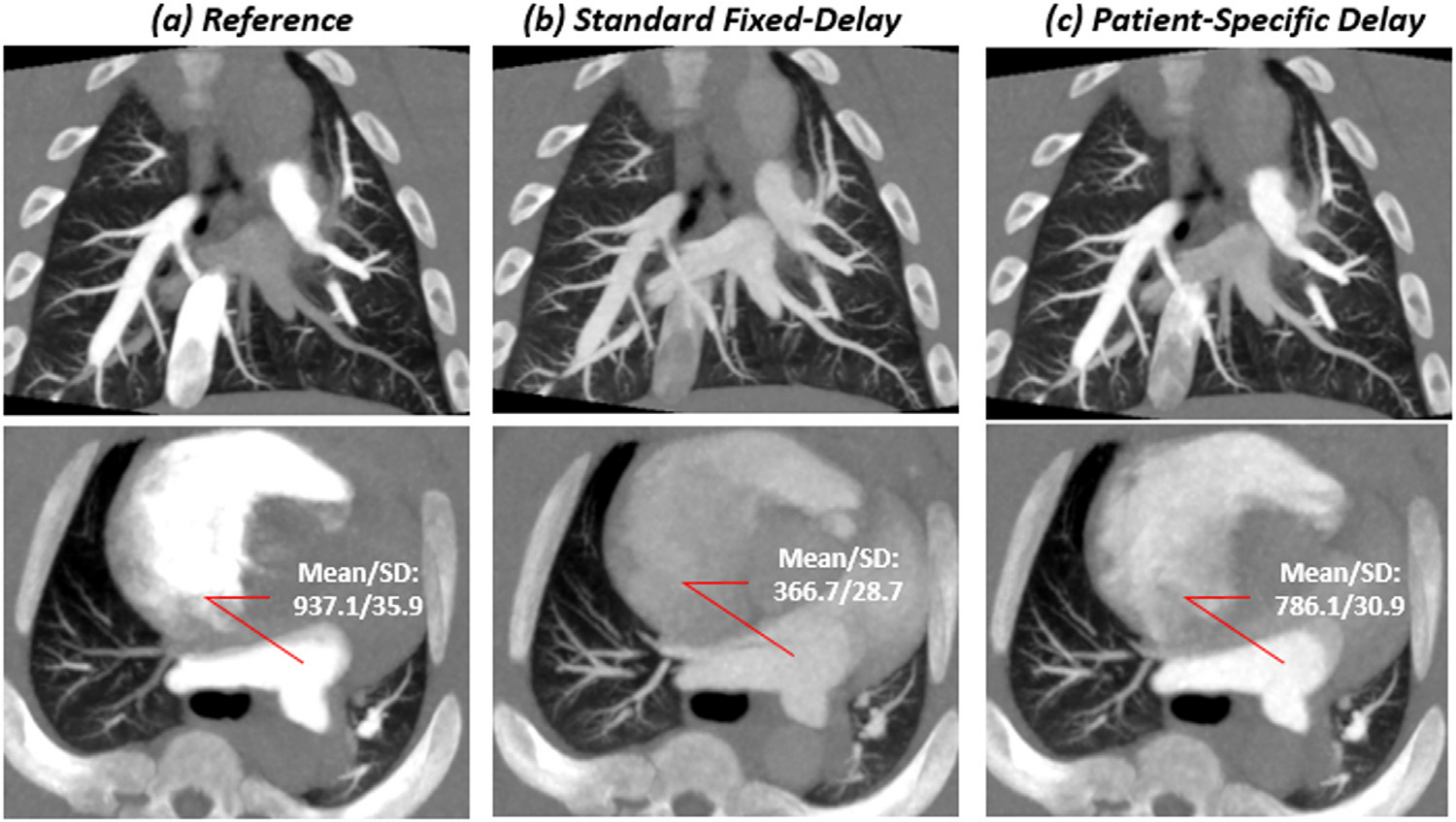
Images for a 55kg swine with an injection of 0.5 ml/kg contrast at a rate of 10 ml/s with no balloon occlusion. Contrast material–enhanced CT angiography (a) using retrospective peak from the pulmonary arterial input function (AIF); (b) with standard fixed-delay protocol; (c) with patient-specific timing protocol in a 10 mm maximum-intensity projection (MIP) image from both coronal and axial rendering ([*window*/*level*: 1600/500]. In the main pulmonary artery, The CT numbers for (a), (b) and (c) were 937.1 HU, 366.7 HU and 786.1 HU; the SNR were 46.8, 18.3 and 39.2; the CNR were 43.4, 14.9 and 35.9, respectively. For subjective image quality the scores were 1, 2, 1 respectively for reader 1, and were 1,3, 1 respectively for reader 2.

**Fig. 4:**
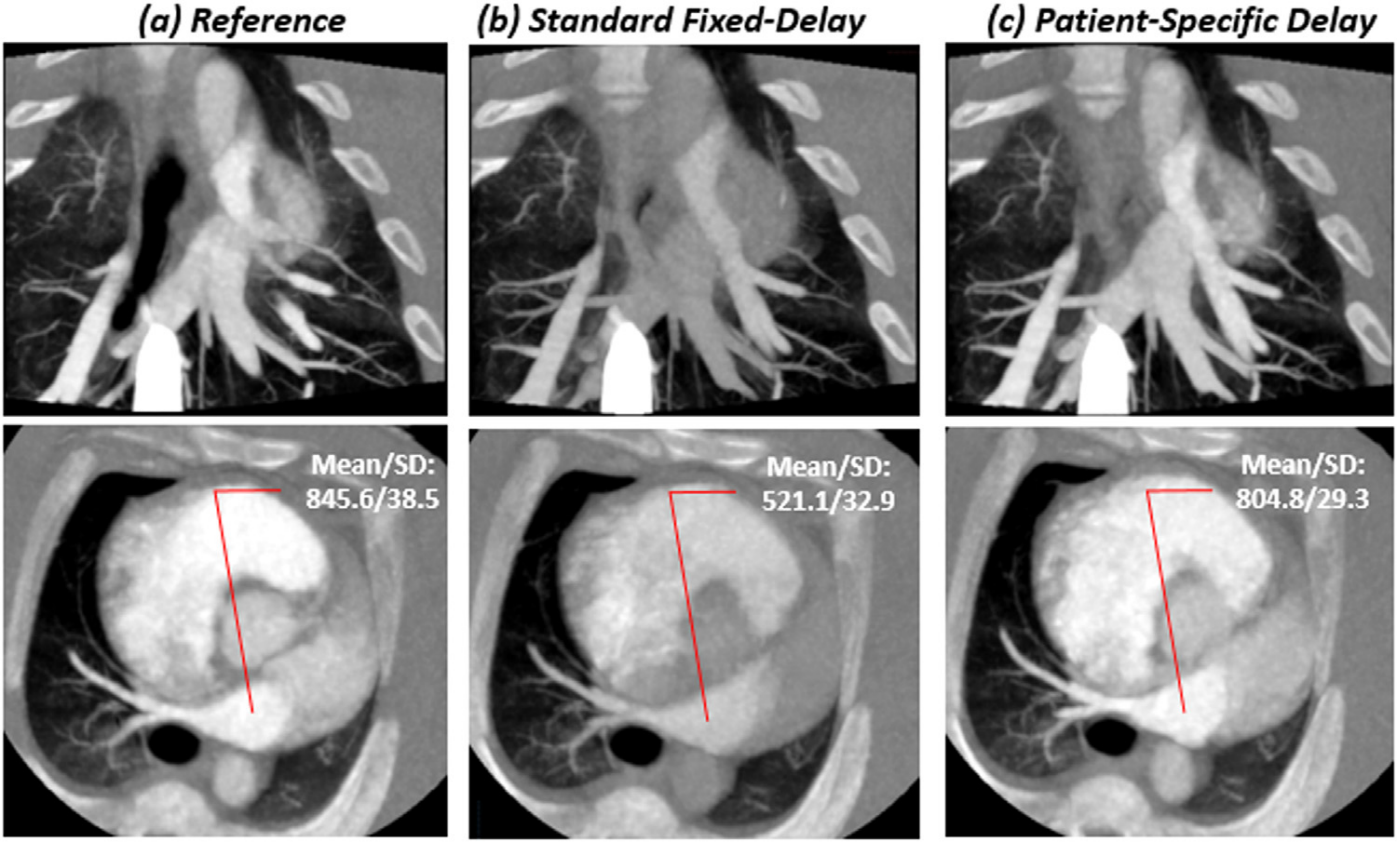
Images for a 91kg swine with an injection of 1 ml/kg contrast at a rate of 5 ml/s with no balloon occlusion. Contrast material–enhanced CT angiography (a) using retrospective peak from the pulmonary arterial input function (AIF); (b) with standard fixed-delay protocol; (c) with patient-specific timing protocol in a 10mm maximum-intensity projection (MIP) image from both coronal and axial rendering ([*window*/*level*: 1600/500]. In the main pulmonary artery, The CT numbers for (a), (b) and (c) were 845.6 HU, 521.1 HU and 804.8 HU; the SNR were 21.8, 13.4 and 20.7; the CNR were 19.6, 11.3 and 18.6, respectively. For subjective image quality the scores were 2, 3, 2 respectively for reader 1 and reader 2.

Overall, a total of 462 CTPA images were assessed (154 images per group) and adequate image quality was achieved in 454 images (98.3%) (Table 1). For group A, all images were rated above adequate quality (100%) for both readers. For group B, the assessment showed excellent quality in 88 of 154 (57.1%), good quality in 50 of 154 (32.5%), adequate quality in 9 of 154 (5.8%), poor quality in 4 of 154 (2.6%), and non-diagnostic quality in and 3 of 154 (1.9%). For group C, the assessment showed excellent quality in 96 of 154 (62.3%), good quality in 45 of 154 (29.2%), adequate quality in 12 of 154 (7.8%), and non-diagnostic quality in only one of 154 (0.6%). Representative examples of CTPA images in three different swine (body weights: 27 kg, 55 kg and 91 kg) from each of study group are provided in Fig. 2, Fig. 3, Fig. 4.

### 3.3 Objective assessment

**Fig. 5:**
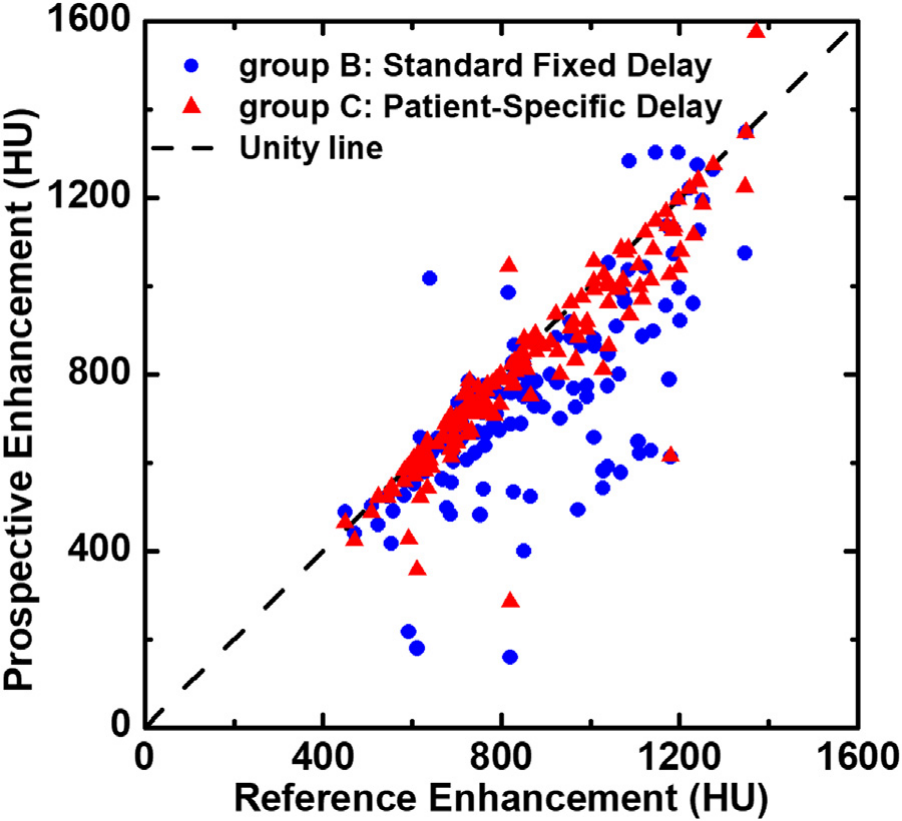
Overall mean contrast enhancements of all measurement locations in pulmonary arteries. Both simulated standard fixed delay (group B) and patient-specific delay (group A) CTPAs are corresponded to the retrospective reference peak (group A). Each point represents a mean contrast enhancement of the main pulmonary artery (MPA), right and left pulmonary artery (RPA, LPA), right upper lobe artery (RUL), right middle lobe artery (RML), right lower lobe artery (RLL), left upper lobe artery (LUL), left iliac artery (LIL), left lower lobe artery (LLL), assessor lobe artery (AL), right lower lobe segmental artery (RLL_S) and left lower lobe segmental artery (LLL_S). The black dash line is the unity line.

**Fig. 6:**
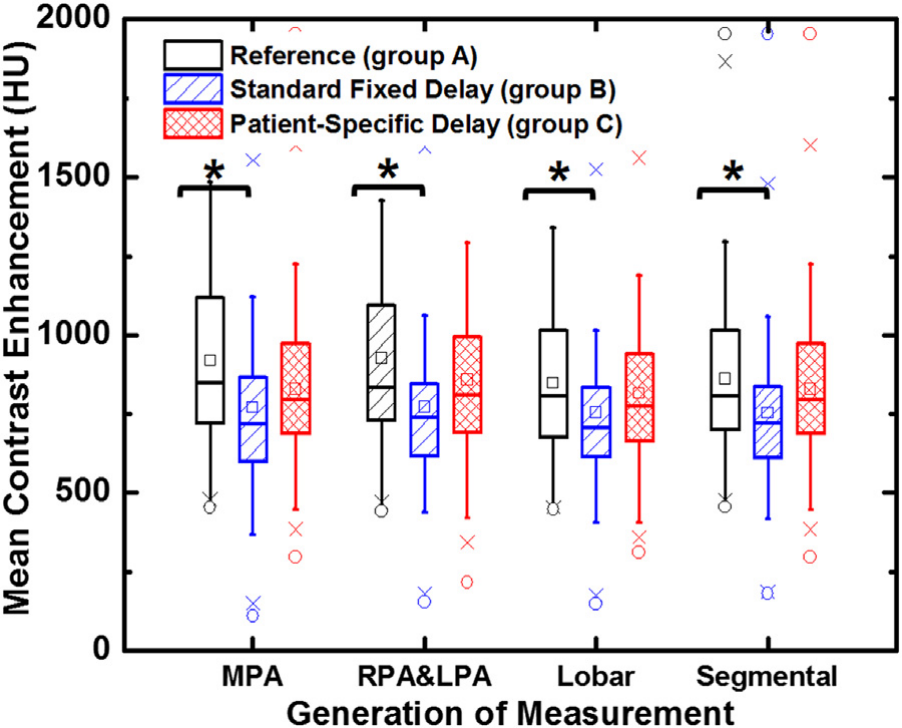
Box plot shows mean contrast enhancements (in Hounsfield units) in three study groups. Note that measurements are averaged within each generation, including the main pulmonary artery (MPA); mean of right and left pulmonary artery (RPA, LPA), mean of lobar artery (RUL, RML, RCL, LUL, LIL, LLL) and mean of segmental arteries (AL, RCL_S and LCL_S). The asterisk indicates the two boxes are significantly different.

For each of CTPA images, the contrast enhancements of 12 pulmonary artery ROIs were measured, resulting in 5424 independent measurements in total. Overall, the mean contrast enhancement of all the pulmonary arteries for group A, B and C were 870 ± 244 HU, 761 ± 247 HU and 826 ± 237 HU, respectively. For each vessel ROI, no significant differences were found between group C and group A (*p* = 0.15), whereas significant differences were found between group B and group A (*p* < 0.001) except for the right caudal lobe artery, as illustrated in Table 2. The mean contrast enhancement of all pulmonary arteries from group B and group C were also compared to group A (reference), as depicted in Fig. 5. In addition, at each pulmonary vessel generation, the differences between the three groups are shown in box plots (Fig. 6). The largest contrast enhancement differences for group B and group C as compared to group A were observed in the right and left main pulmonary artery, calculated as 154 HU (*p* < 0.001) and 67 HU (*p* = 0.055), respectively (Table 3). The smallest contrast enhancement difference between group B and group C was observed in the lobar arteries at 60.6 HU (*p* = 0.025). For overall SNR and CNR, no significant differences were found between group A (SNR: 65.1 ± 32.2; CNR: 59.6 ± 30.8) and group C (SNR: 62.4 ± 32.0, *p* = 0.444; CNR: 56.4 ± 30.3, *p* = 0.421), but significant differences were found between group A and group B (SNR: 56.9 ±3 0.4, *p* = 0.020; CNR: 50.9 ± 28.9, *p* = 0.012) (Table 3). There were no differences in mean muscle contrast enhancement and background noise between groups (Table 3).

**Table 2:** Contrast enhancement from 12 pulmonary arteries subregions.

Location	Group A	Group B			Group C		
(HU)	Mean ± SD [95% CI]	Mean ± SD [95% CI]	Difference	P-values	Mean ± SD [95% CI]	Difference	P-values
MPA	920.1±271.4 [877.6, 962.6]	772.3±277.2 [731.2, 822.0]	−147.8	<0.001	861.0±267.1 [812.4, 903.1]	−59.1	0.058
RPA	918.6±267.4 [871.4, 958.8]	788.6±276.2 [747.9, 837.4]	−130.0	<0.001	876.6±263.6 [835.5, 917.7]	−40.6	0.163
LPA	932.8±569.4 [840.6, 1025.0]	760.9±268.6 [717.4, 804.4]	−171.9	<0.001	836.7±258.5 [794.9, 878.5]	−91.6	0.059
RUL	846.4±251.1 [805.7, 887.0]	760.0±251.5 [719.3, 800.7]	−86.4	0.001	816.8±242.3 [777.5, 856.0]	−29.6	0.290
RML	812.8±224.4 [776.5, 849.1]	740.6±357.6 [682.7, 798.5]	−72.2	0.020	783.5±220.2 [747.9, 819.2]	−29.3	0.247
RCL	887.9±261.6 [845.5, 930.3]	811.3±504.6 [729.6, 893.0]	−76.6	0.062	850.0±257.0 [808.3, 891.5]	−37.9	0.193
LUL	820.0±244.9 [780.3, 859.6]	719.9±239.4 [681.1, 758.7]	−100.1	<0.001	788.2±242.1 [749.0, 827.4]	−31.8	0.260
LIL	831.3±245.0 [791.6, 871.0]	744.1±243.2 [706.1, 782.0]	−87.2	0.001	801.5±238.2 [762.9, 840.1]	−29.8	0.300
LCL	869.4±253.6 [828.3, 910.5]	758.3±261.3 [716.0, 799.8]	−111.1	<0.001	828.0±249.5 [787.6, 868.4]	−41.4	0.157
AL	826.6±241.3 [787.7, 865.4]	723.6±239.8 [684.7, 762.4]	−103.0	<0.001	792.2±231.3 [754.8, 829.7]	−34.4	0.214
RCL_S	871.8±257.8 [830.1, 913.5]	768.5±247.8 [728.4, 808.6]	−103.3	<0.001	833.5±249.5 [793.1, 873.9]	−38.3	0.199
LCL_S	875.1±244.8 [835.4, 914.7]	772.6±250.5 [733.2, 813.0]	−152.5	<0.001	840.5±240.0 [801.7, 879.3]	−34.6	0.225

Note.- Group A: actual peak image from the retrospective arterial input function. Group B: fixed-delay CTPA image. Group C: patient-specific delay CTPA image. Mean signal intensity are shown in mean ± standard deviation at each measurement location. CI = confidence intervals. HU = Hounsfield units. P-values less than 0.05 indicate the significant differences. MPA: main pulmonary artery; RPA: right pulmonary artery; LPA: left pulmonary artery; RUL: right upper lobe artery; RML: right middle lobe artery; RML: right middle lobe artery; RCL: right caudal lobe artery; LUL: left upper lobe artery; LIL: left ligula lobe artery; LCL: left caudal lobe artery; AL: accessory lobe artery; RCL_S: segmental artery in right caudal lobe; LCL_S: segmental artery in left caudal lobe.

**Table 3:** Objective image quality assessments (contrast enhancement, SNR, CNR) at different generations.

Measurement generations		Group A	Group B		Group C	
		(Mean ± SD)	(Mean ± SD)	P-value	(Mean ± SD)	P-value
Overall pulmonary artery	SI (HU)	870.1 ± 244.1	761.1 ± 246.7	< 0.001	825.6 ± 238.8	0.152
	SNR	65.2 ± 32.2	56.9 ± 30.4	0.020	62.4 ± 32.0	0.444
	CNR	59.6 ± 30.8	50.9 ± 28.9	0.012	56.4 ± 30.3	0.421
MPA	SI (HU)	916.5 ± 271.5	776.6 ± 280.2	< 0.001	858.0 ± 279.4	0.058
	SNR	69.2 ± 35.9	57.9 ± 32.7	0.004	64.7 ± 35.3	0.279
	CNR	63.6 ± 34.5	52.3 ± 31.4	0.003	59.1 ± 33.9	0.259
RPA & LPA	SI (HU)	926.7 ± 354.0	773.2 ± 267.3	< 0.001	859.9 ± 265.3	0.055
	SNR	69.2 ± 40.2	57.8 ± 31.6	0.004	64.4 ± 33.2	0.231
	CNR	69.2 ± 35.9	52.2 ± 30.4	0.003	58.5 ± 31.6	0.213
Lobar arteries	SI (HU)	848.5 ± 236.1	756.1 ± 250.0	0.001	816.7 ± 233.1	0.224
	SNR	63.5 ± 31.4	56.6 ± 30.1	0.049	61.4 ± 31.5	0.564
	CNR	57.9 ± 30.0	50.9 ± 28.7	0.039	55.8 ± 30.1	0.543
Segmental arteries	SI (HU)	861.8 ± 239.4	755.8 ± 239.7	< 0.001	828.0 ± 230.0	0.219
	SNR	64.4 ± 31.7	56.5 ± 30.0	0.025	62.2 ± 31.8	0.539
	CNR	58.9 ± 30.3	50.8 ± 28.8	0.011	56.6 ± 30.2	0.513
Muscle	(HU)	75.5 ± 15.5	77.1 ± 10.0	0.152	76.2 ± 11.2	0.277
Noise	(HU)	15.1 ± 5.3	14.7 ± 4.3	0.412	14.1 ± 5.8	0.248

Note.- Group A: actual peak image from the retrospective arterial input function. Group B: fixed-delay CTPA image. Group C: patient-specific delay CTPA image. Mean signal intensity (SI), signal-to-noise ratio (SNR) and the contrast-to-noise ratio (CNR) are shown as mean ± standard deviation. HU = Hounsfield units.

## 4 Discussion

The results indicate that the patient-specific timing protocol for CTPA produces significantly improved image quality as compared with the standard protocol with a fixed time delay between bolus tracking trigger and image acquisition. The improvements were shown by using the objective image metrics including contrast enhancement, CNR and SNR, as well as subjective image quality for pulmonary arterial opacification at all generations. Hence, if the patient-specific protocol can be employed prospectively, there exists potential to improve CTPA image quality or reduced contrast volume as compared to the existing standard CTPA or dual-energy CT protocols.

The patient-specific timing protocol was shown to be robust over a range of cardiac outputs, contrast injection volumes and durations. Such findings also agree with a previous report indicating that the aortic time-to-peak delay is primarily affected by the injection duration [7]. Specifically, given a short injection time (< 15 s) and a fast injection rate (> 4ml/s), the shape of the arterial input function curve is approximately normally distributed prior to the recirculation [7]. Hence, the time-to-peak delay ($t_{ttp}$) can be linearly related to one-half of the total injection time ($\frac{t_{inj}}{2}$) plus a constant dispersion delay ($t_{dis}$) that describes the degree of contrast dispersion occurring between the venous injection site and vessel or organ of interest [25]. The estimated constant dispersion delay for pulmonary artery and descending aorta have been reported to be 1s and 2s, respectively [25,29].

It is also important to point out that the results indicate the potential for contrast volume reduction, while maintaining adequate image quality. A higher than necessary contrast dose is often used for CTPA protocols to ensure adequate contrast opacification is obtained [1,5,6,17,19,30]. However, given the results from this study, a contrast dose of 0.5 ml/kg provided good diagnostic performance for the patient-specific protocol group, as the CTPA acquisition always occurred at or near the peak enhancement. Hence, the patient-specific timing protocol can potentially reduce the contrast dose, although further validation in patients remains necessary.

A fixed threshold is currently used for bolus tracking in the CTPA image acquisition where a suitable triggering threshold is critical for acquisition of peak enhancement. In general, a low threshold may result in false triggering prior to adequate contrast bolus arrival in the pulmonary vasculature, while a high threshold may lead to late acquisition with venous contamination. Further, the bolus tracking threshold varies between different CT scanners and scan protocols. In this study, multiple contrast injections were made in each animal so bolus tracking threshold was calculated for each study by adding 60 HU to the baseline blood pool Hounsfield units.

The successful clinical integration of this timing protocol in the future will necessitate the inclusion of bolus tracking and accurate determination of contrast injection durations for predicting the time to peak enhancement. While validation in patients with varying cardiac outputs is essential, it is anticipated that dispersion delays for the pulmonary artery and descending aorta will also average approximately 1 s and 2 s, respectively. Assuming the adoption of a weight-based injection protocol and a predetermined injection rate, the specific weight of the patient will determine the contrast injection duration and consequently determining the prediction of the time to peak enhancement.

## 5 Limitations

First, all acquisitions were performed in swine, and not in human patients. However, owing to the requirement for capturing the entire contrast pass curve to serve as the reference standard, the delivered high radiation dose is more feasible in animal studies. Second, the ‘prospective’ results were derived based on standard and patient-specific timing protocol simulations performed on the 20 volume scans data that included the entire contrast pass curve. Future studies need to compare the prospective performance of the patient-specific timing protocol to the standard protocol. Further considerations of bolus tracking-based triggering and acquisition should also be addressed. Specifically, the location of the triggering ROI, the scan range, and the standard acquisition delay used by different CT scanners may all need to be considered by the patient-specific timing protocol. Third, objective image quality was only assessed down to segmental arterial generations; hence, more distal arterial generations may need to be assessed in future studies. Fortunately, significant differences were found in all measured vessel locations between patient-specific and fixed delay timing protocols, with higher subjective image quality obtained by the patient-specific timing protocol in the distal vessels. Fourth, radiation dose was not optimized in this study as the focus was to develop and assess a patient-specific timing protocol for improved CTPA contrast opacification. Future studies should employ the patient-specific findings of this study prospectively, at which point radiation dose can truly be assessed. Additionally, the kilovoltage settings should be adjusted according to each patients’ weight in future studies. Finally, the diagnostic performance using the patient-specific timing protocol requires further testing in patients with actual pulmonary pathologies such as the acute pulmonary embolism and chronic thromboembolic pulmonary hypertension.

## 6 Conclusion

The patient-specific timing protocol has the potential to objectively improve contrast opacification in CTPA while also improving subjective image quality. Such improvements in image quality throughout successive pulmonary arterial generations may also improve the diagnostic accuracy and detection of small pulmonary embolisms compared to the standard fixed-delay CTPA protocols.

## Data share statement

Data generated or analyzed during the study are available from the corresponding author by request.

## Ethical statement

The study was approved by the Institutional Animal Care and Use Committee (IACUC, Protocol Number: AUP-18–191) at University of California, Irvine.

## CRediT authorship contribution statement

**Yixiao Zhao:** Conceptualization, Formal analysis, Investigation, Project administration, Software, Supervision, Writing – original draft, Writing – review & editing. **Logan Hubbard:** Conceptualization, Formal analysis, Investigation, Project administration, Software, Supervision, Writing – original draft, Writing – review & editing. **Shant Malkasian:** Conceptualization, Formal analysis, Investigation, Project administration, Software, Supervision, Writing – original draft, Writing – review & editing. **Pablo Abbona:** Conceptualization, Formal analysis, Investigation, Project administration, Software, Supervision, Writing – original draft, Writing – review & editing. **Vijay Bosemani:** Conceptualization, Formal analysis, Investigation, Project administration, Software, Supervision, Writing – original draft, Writing – review & editing. **Sabee Molloi:** Conceptualization, Formal analysis, Investigation, Project administration, Software, Supervision, Writing – original draft, Writing – review & editing.

## Declaration of Competing Interest

The authors declare the following financial interests/personal relationships which may be considered as potential competing interests: Sabee Molloi reports financial support was provided by Canon America Medical Systems.

## References

[1] Schoepf U.J., Costello P. (2004). CT angiography for diagnosis of pulmonary embolism: state of the art. Radiology.

[2] Rémy-Jardin M., Pistolesi M., Goodman L.R., Gefter W.B., Gottschalk A., Mayo J.R. (2007). Management of suspected acute pulmonary embolism in the era of CT angiography: a statement from the fleischner society. Radiology.

[3] Sauter A., Koehler T., Fingerle A.A., Brendel B., Richter V., Rasper M. (2016). Ultra low dose CT pulmonary angiography with iterative reconstruction. PLOS One.

[4] Sharma S., Lucas C.D. (2017). Increasing use of CTPA for the investigation of suspected pulmonary embolism. Postgrad Med.

[5] Moore A.J.E., Wachsmann J., Chamarthy M.R., Panjikaran L., Tanabe Y., Rajiah P. (2018). Imaging of acute pulmonary embolism: an update. Cardiovasc Diagn Ther.

[6] van Es J., Douma R.A., Schreuder S.M., Middeldorp S., Kamphuisen P.W., Gerdes V.E.A. (2013). Clinical impact of findings supporting an alternative diagnosis on CT pulmonary angiography in patients with suspected pulmonary embolism. Chest.

[7] Bae K.T. (2010). Intravenous contrast medium administration and scan timing at CT: considerations and approaches. Radiology.

[8] Hinzpeter R., Eberhard M., Gutjahr R., Reeve K., Pfammatter T., Lachat M. (2019). CT angiography of the aorta: contrast timing by using a fixed versus a patient-specific trigger delay. Radiology.

[9] Fleischmann D., Kamaya A. (2009). Optimal vascular and parenchymal contrast enhancement: the current state of the art. Radiol Clin N Am.

[10] Ng C.S., Chandler A.G., Chen Y., Wei W., Tannir N.M., Hobbs B.P. (2023). Effect of scan duration on CT perfusion values in metastases from renal cell carcinoma. Res Diagn Interv Imaging.

[11] Belkouchi Y., Lederlin M., Ben Afia A., Fabre C., Ferretti G., de Margerie C. (2023). Detection and quantification of pulmonary embolism with artificial intelligence: the SFR 2022 artificial intelligence data challenge. Diagn Interv Imaging.

[12] Higashigaito K., Schmid T., Puippe G., Morsbach F., Lachat M., Seifert B. (2016). CT angiography of the aorta: prospective evaluation of individualized low-volume contrast media protocols. Radiology.

[13] Brenner D.J. (2010). Should we be concerned about the rapid increase in CT usage?. Rev Environ Health.

[14] Patel S., Kazerooni E.A., Cascade P.N. (2003). Pulmonary embolism: optimization of small pulmonary artery visualization at multi-detector row CT. Radiology.

[15] Schoellnast H., Deutschmann H.A., Fritz G.A., Stessel U., Schaffler G.J., Tillich M. (2005). MDCT angiography of the pulmonary arteries: influence of iodine flow concentration on vessel attenuation and visualization. AJR Am J Roentgenol.

[16] Nijhof W.H., Jansen M.M., Jager G.J., Slump C.H., Rutten M.J. (2016). Feasibility of a low concentration test bolus in CT angiography. Clin Radiol.

[17] Moradi M., Khalili B. (2016). Qualitative indices and enhancement rate of CT pulmonary angiography in patients with suspected pulmonary embolism: comparison between test bolus and bolus-tracking methods. Adv Biomed Res.

[18] Hubbard L., Malkasian S., Zhao Y., Abbona P., Molloi S. (2019). Timing optimization of low-dose first-pass analysis dynamic CT myocardial perfusion measurement: validation in a swine model. Eur Radiol Exp.

[19] Wang M., Li W., Lun-Hou D., Li J., Zhai R. (2015). Optimizing computed tomography pulmonary angiography using right atrium bolus monitoring combined with spontaneous respiration. Eur Radiol.

[20] Kai N., Oda S., Utsunomiya D., Nakaura T., Funama Y., Kidoh M. (2018). Dual-region-of-interest bolus-tracking technique for coronary computed tomographic angiography on a 320-row scanner: reduction in the interpatient variability of arterial contrast enhancement. Br J Radiol.

[21] Cao L., Liu X., Li J., Liang W., Qu T., Li Y. (2019). Improving the degree and uniformity of enhancement in coronary CT angiography with a new bolus tracking method enabled by free breathing. Acad Radiol.

[22] Yoshida M., Matsumoto Y., Masuda T., Kikuhara Y., Kobayashi Y., Yoshiura T. (2020). [Comparison of contrast enhancement between bolus-tracking and test-bolus methods on coronary CT angiography]. Nihon Hoshasen Gijutsu Gakkai Zasshi.

[23] Cademartiri F., Nieman K., van der Lugt A., Raaijmakers R.H., Mollet N., Pattynama P.M. (2004). Intravenous contrast material administration at 16-detector row helical CT coronary angiography: test bolus versus bolus-tracking technique. Radiology.

[24] Cademartiri F., Van Der Lugt A., Luccichenti G., Pavone P., Krestin G.P. (2002). Parameters affecting bolus geometry in CTA: a review. J Comput Assist Tomogr.

[25] Zhao Y., Hubbard L., Malkasian S., Abbona P., Molloi S. (2022). Contrast timing optimization of a two-volume dynamic CT pulmonary perfusion technique. Sci Rep.

[26] Moore B.M., Brady S.L., Mirro A.E., Kaufman R.A. (2014). Size-specific dose estimate (SSDE) provides a simple method to calculate organ dose for pediatric CT examinations. Med Phys.

[27] Schauer D.A., Linton O.W. (2009). Ionizing radiation exposure of the population of the united states, medical exposure-are we doing less with more, and is there a role for health physicists?. Health Phys.

[28] Zhao Y., Hubbard L., Malkasian S., Abbona P., Molloi S. (2020). Dynamic pulmonary CT perfusion using first-pass analysis technique with only two volume scans: validation in a swine model. PLOS One.

[29] Hubbard L., Malkasian S., Zhao Y., Abbona P., Molloi S. (2019). Timing optimization of low-dose first-pass analysis dynamic CT myocardial perfusion measurement: validation in a swine model. Eur Radiol Exp.

[30] Rodrigues J.C., Joshi D., Lyen S.M., Negus I.S., Manghat N.E., Hamilton M.C. (2014). Tube potential can be lowered to 80 kVp in test bolus phase of CT coronary angiography (CTCA) and CT pulmonary angiography (CTPA) to save dose without compromising diagnostic quality. Eur Radiol.

